# Evaluation of Two‐Body Wear of Nanocomposites With Different Filler Morphology and Composition Using White‐Light Interferometry

**DOI:** 10.1155/ijod/8929009

**Published:** 2026-02-01

**Authors:** Renáta Martos, Ágnes Szokol, Miklós Veres, József Gáll, Attila Csík, Csaba Hegedűs, Andrea Keczánné-Üveges, Enikő Rita Tóth, Melinda Szalóki

**Affiliations:** ^1^ Department of Operative Dentistry and Endodontics, Faculty of Dentistry, University of Debrecen, Debrecen, Hungary, unideb.hu; ^2^ HUN-REN Wigner Research Center for Physics, Budapest, Hungary; ^3^ Institute of Physics, Faculty of Sciences, University of Pécs, Pécs, Hungary, pte.hu; ^4^ Department of Applied Mathematics and Probability Theory, Faculty of Informatics, University of Debrecen, Debrecen, Hungary, unideb.hu; ^5^ HUN-REN Institute for Nuclear Research Debrecen, Debrecen, Hungary; ^6^ Department of Biomaterials and Prosthetic Dentistry, Faculty of Dentistry, University of Debrecen, Debrecen, Hungary, unideb.hu

**Keywords:** nanocomposites, particulate fillers, two-body wear, white-light interferometry

## Abstract

**Objectives:**

The aim was to compare the two‐body wear behavior of four nanocomposites used for enamel replacement.

**Materials and Methods:**

Nanocomposite specimens (Estelite Asteria [EA], Enamel Biofunction [EBF], Neospectra [NS], Clearfil Majesty [CM]; *n* = 8 for each, diameter = 10 mm, height = 1.5–2 mm) were prepared in a custom‐made mold according to the manufacturer’s instructions. The degree of conversion (DC) was measured by Fourier transform infrared spectroscopy. Vickers hardness (VH) was measured on the top and bottom surfaces, and the VH ratio (VHR) was calculated. The specimens were aged using a thermocycling machine (10,000 cycles), followed by 120,000 chewing cycles. The mean volume loss (MVL), maximum wear depth (MWD), and surface roughness (SR) were assessed with a white‐light interferometer. The tested surfaces before and after the wear test, along with the morphology of extracted fillers, were evaluated using scanning electron microscopy (SEM). Statistical analysis—ANOVA, Levene, Tukey, and Tamhane tests—was performed with SPSS Statistics version 28.

**Results:**

CM exhibited a significantly higher VH compared to the other nanocomposites, both before and after aging. It also showed lower MVL, MWD, and SR than the other three tested nanocomposites.

**Conclusions:**

VH and wear behavior are significantly affected by the filler parameters of nanocomposites.

**Clinical Significance:**

This study may assist clinicians in selecting resin‐based composite (RBC) for occlusal rehabilitation. Based on this in vitro study, CM exhibited a lower wear rate than the other tested RBCs; therefore, it is worth considering its use for patients with higher bite forces.

## 1. Introduction

Wear in the oral cavity is a physical process involving contact between opposing teeth resulting from hitting and sliding movements. Broadly, the underlying mechanisms can be adhesive, abrasive, fatigue, or corrosive wear, depending on the type of interaction. When wear leads to the loss of tooth structure or restorative material, it may cause both functional and esthetic disturbance; restoring the ideal surface shape becomes crucial [1, 2]. Finding the ideal restorative material is a critical task influenced by patient‐, operator‐, and material‐dependent factors [3]. Resin‐based composites (RBCs) are the first‐choice restorative material used in clinical practice. However, RBC subgroups differ in their chemical composition and physical properties, which are associated with clinical performance, such as wear [4–7]. Indeed, their long‐term wear resistance in the posterior region remains controversial.

Nanohybrid and nanofill composites are marketed as versatile additive restorative materials for both anterior and posterior teeth. These RBCs exhibit excellent mechanical properties, polishability, and high esthetics due to the presence of a high ratio of micro‐ and nanoscale fillers within various resin matrices. In contrast, their in vivo wear behavior still raises questions [5, 8].

Wear is a complex process with multiple contributing factors and mechanisms. Therefore, predicting the wear properties of heterogeneous nanocomposites is challenging [1, 9]. Based on previous findings, the filler technology, filler load, and resin composition affect the basic characteristics of RBCs [5, 10, 11]. Researchers have observed links between certain laboratory parameters and clinical performance, but due to the large number of variable factors, the results are inconclusive [6, 12]. The complex individual microstructure significantly influences physical properties of RBCs, including the surface Vickers hardness (VH), which correlates with surface mechanical properties and directly influences in vivo performance, such as wear resistance [8, 13–15]. Furthermore, the VH ratio (VHR) of an RBC can serve as an indicator of the monomer conversion rate [16].

In vitro testing of wear parameters involves the use of simulators and wear methods [17]. Besides simplifying the real clinical environment, these approaches mimic various aspects of the oral environment, such as bite force, sliding movement, sliding distance, and the radius size of the antagonist teeth. The two‐body wear testing methods replicate an isolated and well‐controlled setting, allowing researchers to compare materials and evaluate the wear of restorative materials [18, 19]. In this in vitro test, the tested material is in direct contact with the antagonist material. Artificial aging in a thermocycler simulates both a wet environment and thermal stress. These effects frequently occur in the oral cavity and can lead to deterioration of the physical properties of the RBCs [12].

In this study, we aimed to evaluate the conversion, VH, surface roughness (SR), and two‐body wear resistance of four nanocomposites for enamel replacement after thermocycling in a Willytec chewing simulator using a steatite antagonist. The nanocomposite group exhibits significant variation in filler size, shape, and distribution, embedded within different matrix compositions [9]. Furthermore, the quality and strength of the bond between the fillers and the matrix, as well as the microstructure’s function influence the long‐term stability of the surface [20, 21]. For this reason, we selected our tested composites to include homogeneously distributed monomodal spherical fillers, inhomogeneously distributed prepolymer fillers combined with particulate fillers, or inhomogeneously distributed particulate fillers of various sizes, with or without agglomerated nanoparticles in different resin matrix compositions.

Our null hypotheses are the following:

1. There is no significant difference in two‐body wear resistance of nanocomposites with different compositions and microstructure.
2. There is no association between VH, filler technology, and wear behavior of nanocomposites with different compositions and microstructure.

## 2. Materials and Methods

### 2.1. Specimen Preparation

Eight cylindrical specimens were prepared (diameter = 8 mm, height = 1.5 or 2 mm as a suggested maximum layer thickness) according to the manufacturer’s instructions in a three‐dimensionally (3D)‐printed resin mold (MED 620 Biocompatible A2 resin, Stratasys, Eden Prairie, MN USA; Figure 1). Four nanocomposites were examined: Estelite Asteria (EA), Enamel Biofunction (EBF), Neospectra (NS), and Clearfil Majesty (CM). The composition, maximum layer thickness, and additional information of the materials used in the study are shown in Table 1. A glass microscope slide was fixed on the bottom and top of the flat and standardized specimens to achieve maximum smoothness on the top and bottom surfaces. The polymerization protocol was a high‐mode curing program using Bluephase 20i (Ivoclar, Schaan, Lichtenstein) at 1200 mW/cm^2^ for 20–40 s, according to the manufacturer’s instructions, followed by storage at room temperature for 24 h. A digital radiometer was used to monitor the average irradiance of the light source before and after irradiation (1200 mW/cm^2^). The top surface of the specimens was marked with a permanent marker. Polishing was not applied on the top and bottom surfaces.

Figure 1The three‐dimensionally printed resin molds for specimen preparation with a thickness of (a) 2 mm and (b) 1.5 mm.

**Figure:**
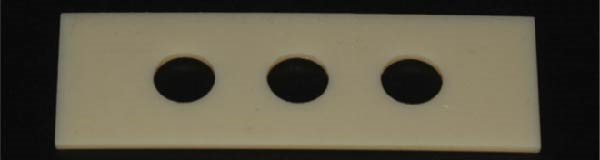
(a)

**Figure:**
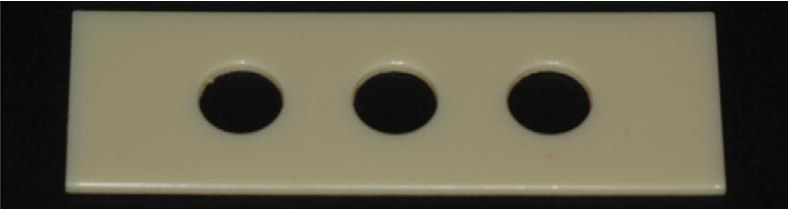
(b)

**Table 1:** Details on the composition, maximum layer thickness, curing time, and other information about the materials used in this study.

Name (manufacturer) abbreviation	Monomers	Fillers	LOT number maximum layer thickness	Curing time (s)
Estelite Asteria OcE (Tokuyama dental corporation, Japan) Code: EA	Bis‐GMA, Bis‐MPEPP, UDMA, TEGDMA	Spherical SiO_2_–ZrO_2_ 0.2 μm 82 wt% (71 vol%)	W9245 1.5 mm	20
Enamel Plus Hri Bio‐Function (Micerium, Avegno Genova, Italy) Code: EBF	UDMA, tricyclodecae dimethanol dimethacrylate	Aggregated 5–50 nm SiO_2_; 0.2–3 µm glassy particles 74 wt% (60 vol%)	2019008149 2 mm	40
Neospectra (Dentsply Konstanz, Germany) Code: NS	Methacrylic modified polysiloxane, (organically modified ceramic), dimethacrylate resins, ethyl 4 (dimethylamino)benzoate, bis(4‐methyl‐phenyl) iodonium, hexafluorophosphate	Barium glass, ytterbium fluoride, prepolymerized sphere TEC fillers 0.1–0.3 μm, mean 0.2 μm 78–80 wt% (60–62 vol%)	1908001068 1.5 mm	20
Clearfil Majesty (Kuraray Noritake, Tokyo, Japan) Code: CM	Bis‐GMA, TEGDMA, hydrophobic aromatic dimethacrylate	Glass ceramics, pulverized glass filler, and alumina nanofiller 1.5 µm, 20 nm 92 wt% (82 vol%)	B50076 2 mm	20

### 2.2. Measurement and Calculation of the Degree of Conversion (DC)

Fourier transform infrared spectroscopy was performed with a Nicolet 6700 instrument (Thermo Electron Co., Madison WI, USA) to determine the DC of the nanocomposite specimens in the attenuated total reflectance (ATR) mode. The applied wavelength range was 650–4000 cm^−1^. Eight specimens from each group were measured three times on the top surfaces. The top surface is closest to the polymerization light source. One measured point means an average of 16 spectra or 16 measurements. The DC was calculated from the ratio of the double‐bond conversion content of the monomer to the polymer with the following formula [22]:

DC %=Apolymer aliphaticApolymer internal standardAmonomer aliphaticAmonomer internal standard×100,

where *A*_polymer aliphatic_ is the absorbance of the aliphatic group (C═C) of the polymer (peak at 1637 cm^−1^), *A*_polymer internal standard_ is the absorbance of the internal standard (aromatic C═C) in the polymer (peak at 1610 cm^−1^), *A*_monomer aliphatic_ is the absorbance of the aliphatic group (C═C) of the monomer mixture before polymerization, and A_monomer internal standard_ is the absorbance of the internal standard (aromatic C═C) in the monomer before polymerization. For EBF, the internal standard was the carboxylic functional group (C═O) because this nano‐hybrid composite does not contain Bis‐GMA [23, 24].

### 2.3. Measurement of VH and Calculation of VHR

After 24 h of storage, VH was measured using an HV‐120 instrument (Mitutoyo, Kanagawa, Japan) before and after thermocycling. Each specimen was measured five times, randomly at 1 mm from the center point on the top and bottom surfaces, using a diamond 136° pyramid indenter. The applied load was 300 g for 10 s at each measurement point. The VHR was calculated based on the VH of the bottom and top surfaces.

VHR=VHbottom surfaceVHtop surface×100.

### 2.4. Aging of the Specimens

After measuring VH, the specimens were aged in a THE‐1200 thermocycler (SD Mechatronik GmbH, Feldkirchen‐Westerham, Germany). Specifically, each specimen was subjected to 10,000 cycles at 5–55°C, with a 30 s dwell time [25].

### 2.5. Two‐Body Wear Test

After thermocycling, a Willytec chewing simulator (SD Mechatronik, Munich Germany) was used for the two‐body wear test. The specimens were mounted in the test chamber, which was filled with distilled water. One of each of the four samples was built and tested simultaneously. The setting parameters were: a loading force of 50 kg (accepted as 49 N), lateral movement of 0.7 mm, a descending speed of 40 mm/s, a lifting speed of 50 mm/s, a frequency of 2.1 Hz, a height of 2 mm, and 120,000 cycles [18].

### 2.6. Measurements of the Mean Volume Loss (MVL), the Maximum Wear Depth (MWD), and SR

**Figure 2:**
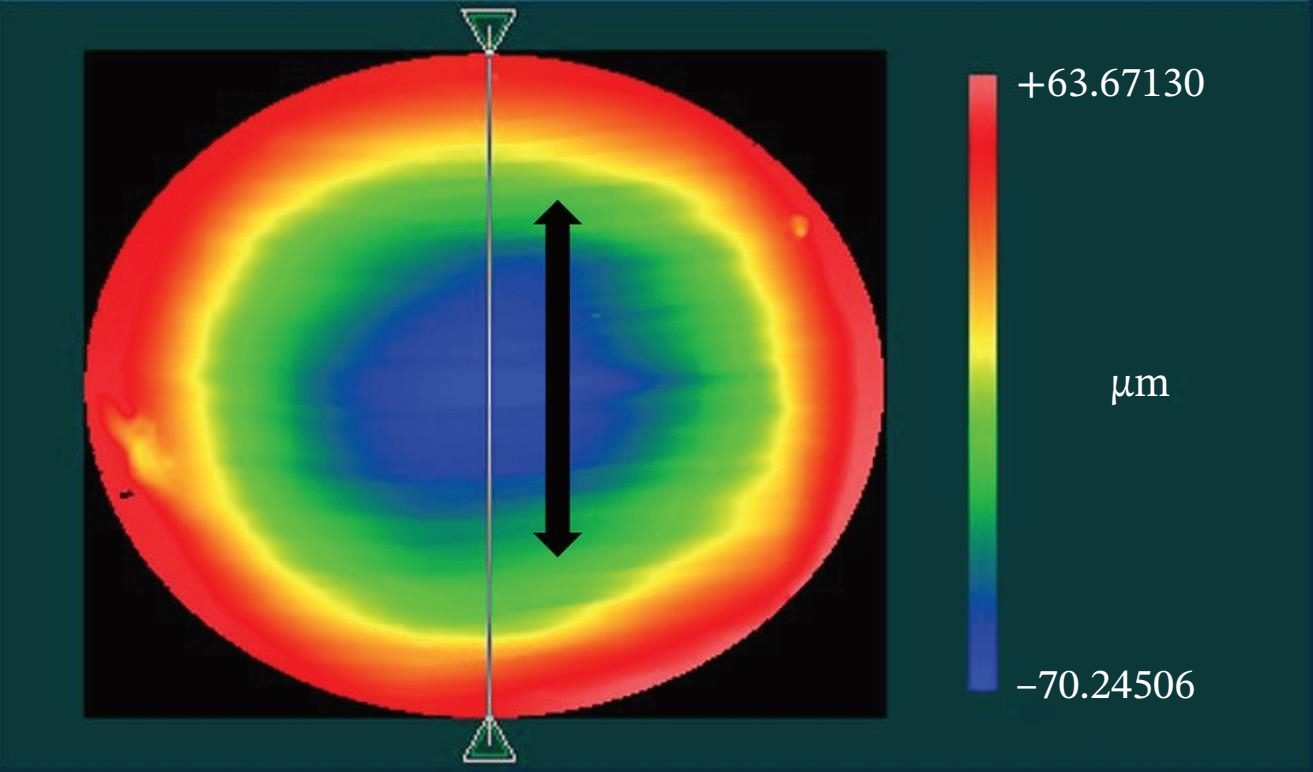
The *y*‐axis was defined as the plane perpendicular to the antagonist movement along the entire wear scar. The black arrow shows the line of the measurement (white‐light interferometer).

A white‐light interferometer with a Mirau objective lens (Zygo Corporation, Middlefield, Ohio, USA) was used for imaging at 10× magnification and a field of view of 0.94 mm × 0.7 mm. Two scanning lengths were applied according to the depth of the worn‐out region, namely, under and over 150 µm with vertical resolutions of 0.1 and 20 nm, respectively. Because the investigated area exceeded the objective lens’s field of view, the stitching method was applied, to maintain the same resolution across the entire region of interest. Thus, the interferometer performed 4 × 4 or 5 × 4 measurements in a snake‐line pattern, and the software subsequently stitched them together into a single image with 25%–30% overlap. The sample was moved with a motorized stage during the stitching, but the stage was manually tilted in two directions. Before additional measurements were made, the raw data were preprocessed using low‐pass filtering (taking the median of 21 points for each measurement point) and the built‐in plane removal function. The distance was measured by using the built‐in tool of the instrument software. MVL (worn‐out material, presented in nm^3^) was determined by using a reference plane aligned to the horizontally leveled surface of the sample. The profile characteristics in different cross sections of the tested nanocomposites along the *y*‐axis direction (gray line) were detected; the black arrow in Figure 2 shows the line of the measurement. The mean MWD (in µm) was calculated based on three manual depth measurements in the *x*‐ and *y*‐axes. The SR (presented as the root mean square [RMS, Ra]) from the deepest position of the wear scar was calculated by the instrument as the deviation from the average measured height from the mean linear surface.

### 2.7. Extraction of the Filler Content of Each Tested RBC

A small amount (~0.5 g) of the tested composite was placed into a 15 mL centrifuge tube containing acetone. The mixture was vortexed until all material was dispersed. The extract was centrifuged at 5000 g for 2 min. The supernatants were decanted and the filler suspensions were washed three times with acetone and re‐centrifuged at high speed. The suspensions were dried at room temperature for 24 h [26].

### 2.8. Scanning Electron Microscopy (SEM) Examination

The surface morphology of the samples was examined using a Thermo Scios 2 DualBeam scanning electron microscope (FIB‐SEM, Waltham, MA, USA), equipped with a Bruker Quantax energy dispersive X‐ray spectroscopy (EDS) system to analyze the composition. The analysis was conducted on the treated side of the samples, which were fixed to the sample holder using conductive double‐sided adhesive tape. An accelerating voltage of 10 kV and a beam current of 0.2 nA were applied during the measurements. Because the samples are insulators, they were coated with an approximately 15 nm thick gold layer for the study of surface morphology and microstructure, using an E5000C sputter coater (Bio‐Rad Laboratories GmbH, Munich, Germany) to prevent charge buildup on the surface. This coating enabled the use of higher accelerating voltages to produce sharper images. Before coating, composition analysis was performed for each sample type to prevent overlap of the gold peak with other elements in the energy spectra. Diameters of the filler particles were measured from SEM micrographs using ImageJ software, with at least 100 particles measured per sample.

### 2.9. Statistical Analysis

We have compared the mean values of variables DC, VH, MVL, MWD, and SR, respectively, among the composites. For this, first ANOVA tests—one‐way ANOVA F and the (robust) Welch one—were applied depending on the homogeneity of variances, where the latter question was analyzed by the Levene test. For the Levene test the significance level we used was 5% in all cases. Second, post hoc tests—either Tukey following ANOVA or Tamhane following Welch’s ANOVA—tests were run for the multiple (pairwise) comparison, as well as two‐sample *t*‐tests (including Welch *t*‐test if needed) were used for additional analysis in case of the MWD and SR measurements. Before and after aging mean values were compared by paired (matched) *t*‐tests (*α* = 0.05). All hypothesis tests and descriptives were calculated in IBM SPSS Statistics version 28 (IBM Corp., Armonk, NY, USA), whereas figures of boxplots are made by the ggplot2 package of R, hence, boxplots are based on the statistics of “boxplot‐stat()” function [27]. The statistical calculations of the filler particles’ average diameters and standard deviation were performed using the Microsoft Office Excel program.

## 3. Results

### 3.1. DC

**Figure 3:**
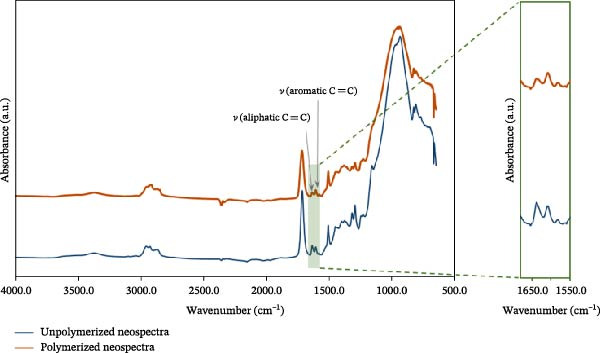
Representative FTIR spectrum of the unpolymerized and polymerized composite (NS; before thermocycling), illustrating the basis of degree of conversion calculation. For clarity, the polymerized FTIR spectrum was vertically offset by +0.3 absorbance units. The characteristic absorption bands used for the analysis are indicated.

Figure 3 illustrates a representative FTIR spectrum of the unpolymerized and polymerized composite (NS) recorded before and after polymerization (before thermocycling). The spectral changes underlying the DC calculation.

**Figure 4:**
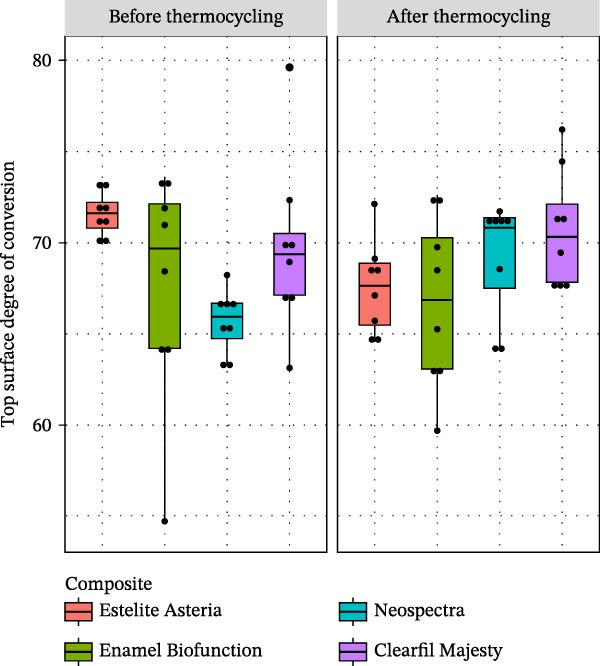
Boxplots of the degree of conversion on the top surface of the tested composites before and after thermocycling (dots show the individual measurements in %).

The distributions of the top surface DC are shown in Figure 4. The mean DC ranged from 65.67% (standard deviation 1.72%) to 71.58% (standard deviation 1.19%) before thermocycling. After thermocycling, the mean DC was between 66.72 (with a standard deviation of 4.70%) and 70.71 (with a standard deviation of 3.26%). Before thermocycling, the DC differed significantly between the EA and NS groups (Welch’s ANOVA, *p*  < 0.001, Tamhane, *p*  < 0.001). After thermocycling, however, the DC did not differ among the composites (ANOVA, *p* = 0.134). Thermocycling resulted in significant changes in the top surface DC, as evidenced by the EA (paired *t*, *p* = 0.009) and NS composites (paired *t*, *p* = 0.001).

### 3.2. VH and VHR

**Figure 5:**
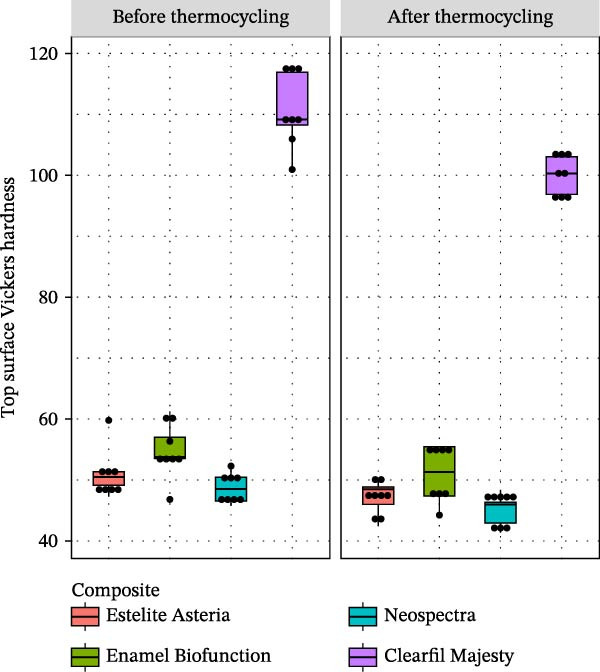
Boxplots of Vickers hardness of the top surface of the tested composites before and after thermocycling (dots show the individual observations).

The distributions of VH are presented in Figure 5. The measurement of VH resulted in significant differences in mean values between CM and any other tested composite both on top and bottom surfaces, as well as both before and after thermocycling (Tamhane for the after‐top case, Tukey for the other three cases gave *p*‐values under 0.001 for all CM vs. other pairs). Thermocycling significantly reduced (mean) VH of the top and bottom surfaces for the EA (paired *t*, *p* = 0.012 and 0.021, respectively), CM (paired *t*, *p*  < 0.001 for both), and NS (paired *t*, *p*  < 0.001 for both) composites, whereas for the EBF case, the reduction was significant only on the top surface (paired *t*, *p* = 0.009).

**Figure 6:**
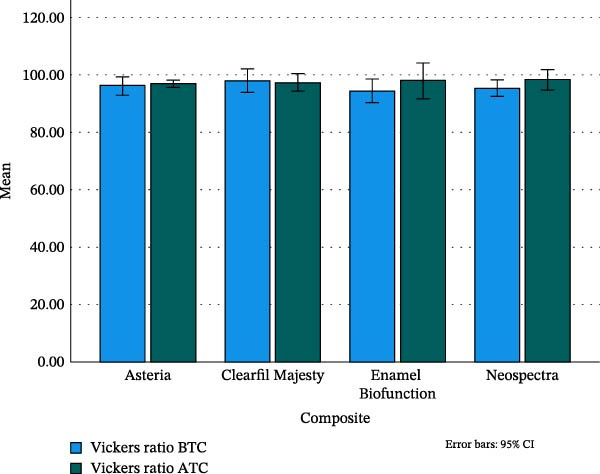
The mean of Vickers hardness ratio of the tested composites in % before and after thermocycling, bars show the 95% CI for the mean. ATC, after thermocycling; BTC, before thermocycling.

There were no significant differences in VHR between the tested composites, neither before (ANOVA, *p*  > 0.402) nor after thermocycling (ANOVA, *p*  > 0.937). Thermocycling did not change the (mean) VHR for the EA, CM, and EBF cases (paired *t*, *p* = 0.531, 0.726, and 0.059, respectively), whereas composite NS showed significantly smaller mean VHR before aging (*p* = 0.030). See Figure 6 for the mean VHR values.

### 3.3. MVL

**Figure 7:**
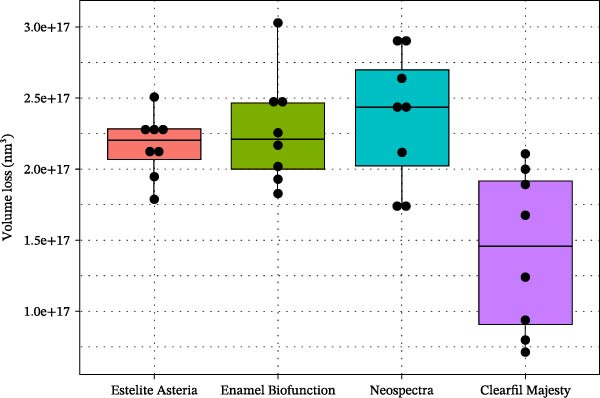
Boxplots of the mean volume loss (nm^3^) of the tested composites (dots show the individual observations).

MVL distributions are shown in Figure 7. The CM case showed a significantly lower (mean) MVL compared with the other composites at level 5% (Welch’s ANOVA, *p* = 0.016, and Tamhane test gave *p* = 0.041, 0.024, and 0.017 vs. EA, EBF, and NS, respectively). The 3D reconstructions of the wear scar in Figure 8 show the surface profiles of the wear surface of the tested composites. The colored lines show the profile characteristics in different cross‐sections perpendicular to the antagonist movement (*y*‐axis) along the whole wear scar.

Figure 8Subfigure (a) shows the three‐dimensional reconstructions of the wear scar of the tested composites (analyzed with a white‐light interferometer) and the surface profiles of the wear scar of the tested composites. The colored lines in subfigure (b) show the profile characteristics of the tested composites in different cross‐sections along the *y*‐axis.

**Figure:**
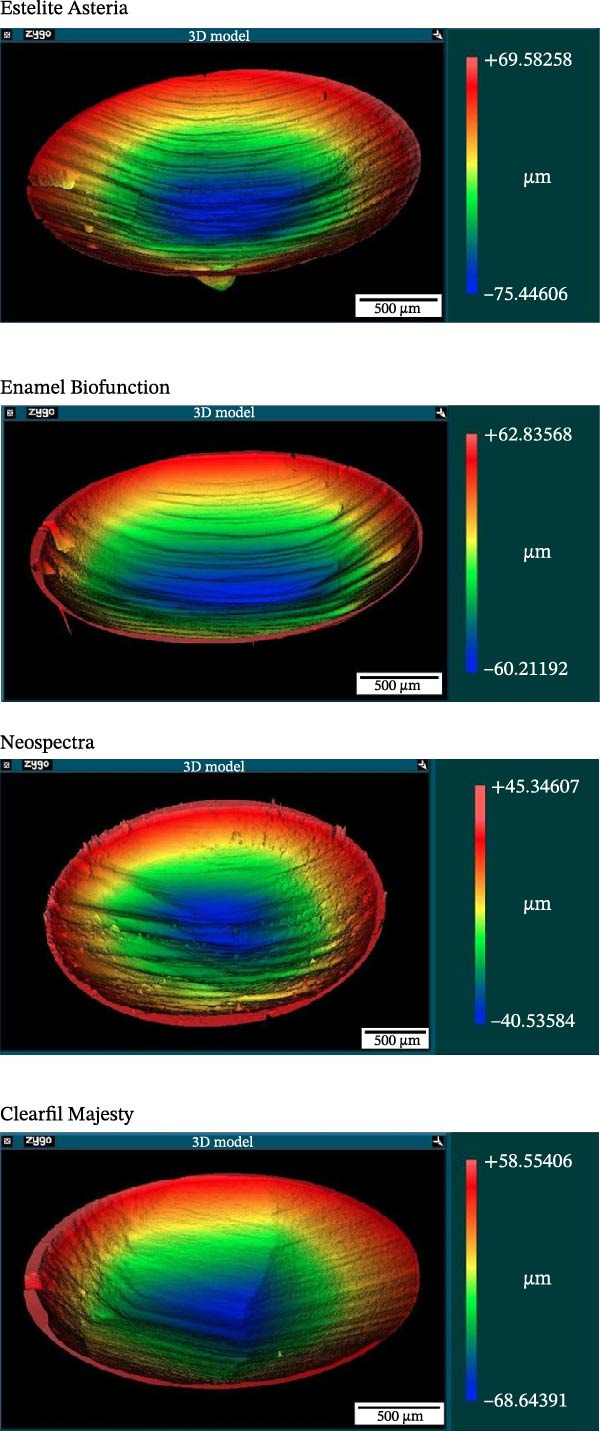
(a)

**Figure:**
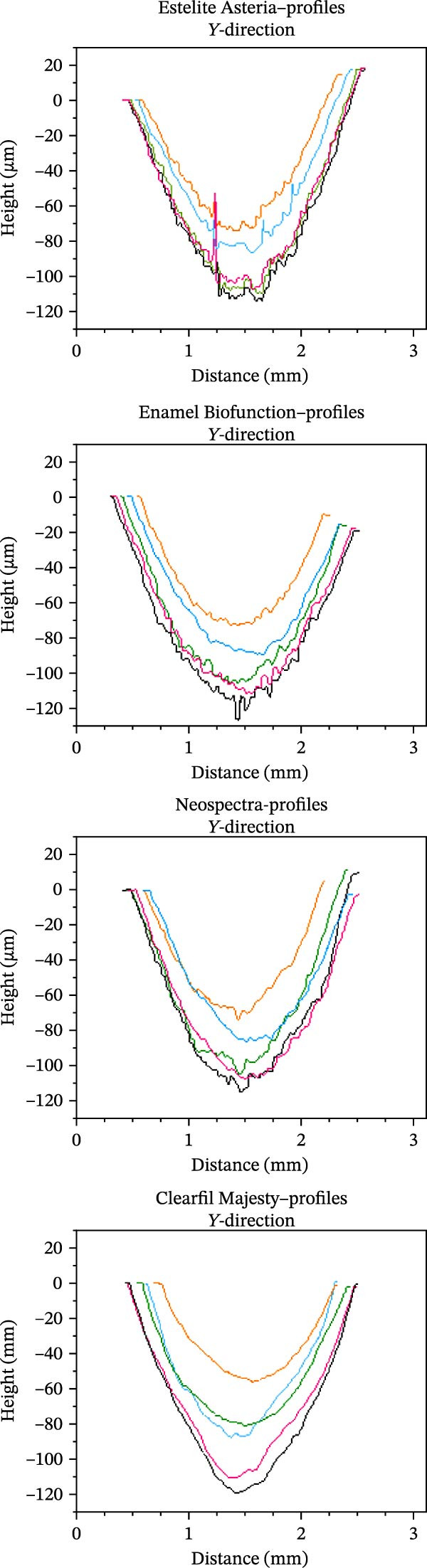
(b)

### 3.4. MWD

**Figure 9:**
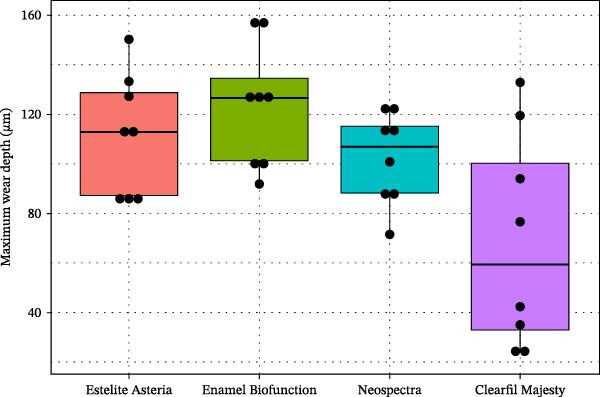
Boxplots of the maximum wear depth (µm) of the tested composites nanocomposites (dots show the individual observations).

MWD data showed a similar pattern (Figure 9) compared to the other three composites as in the case of MVL, that is, CM gave a much lower mean than the others. However, due to larger variances, the difference is found to be significant only compared to the EBF composite (Welch’s ANOVA, *p* = 0.053, Tamhane, *p* = 0.58); whereas an alternative analysis with two‐sample *t*‐tests showed significant differences between CM and any other composite, namely, in cases CM vs. EA (*p* = 0.032) or EBF (*p* = 0.010) at a significance level of 5%, and for the CM vs. NS case at level 10% (*p* = 0.071). Hence, this statistical analysis suggests that a larger sample would likely reveal significant differences between the CM and the other composites when analyzed with ANOVA tests followed by the post hoc tests.

### 3.5. SR

**Figure 10:**
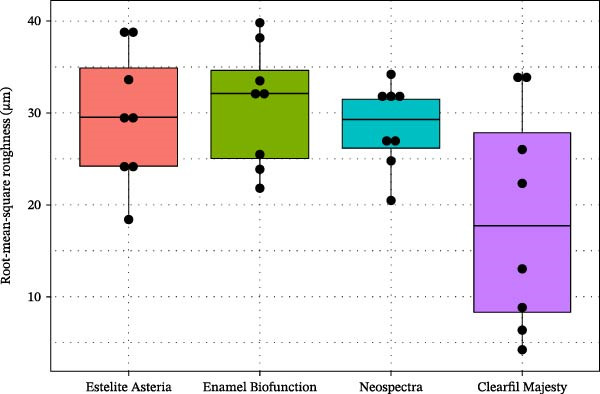
Boxplots of surface roughness (µm) of the tested nanocomposites (dots show the individual observations).

SR measurements also provided a similar pattern, that is, with lower mean SR of CM than in the case of the other composites (Figure 10). However, again due to the large variances—which increases the *p* values in general—these differences were only significant when analyzed by the two sample *t*‐tests, namely, (Welch’s ANOVA, *p* = 0.153), namely, there was a significant difference of mean SR values by the t test between the CM and EA or EBF composites at level 5% (*p* = 0.047 and 0.028), and between the CM and the NS composites at level 10% (*p* = 0.056).

### 3.6. Filler Size and Morphology

**Figure 11:**
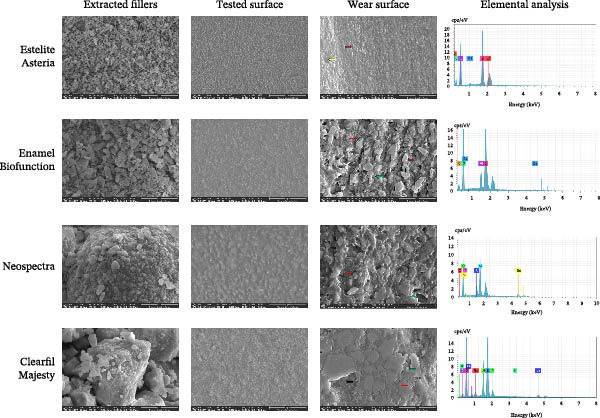
Scanning electron microscope images of the extracted fillers from the tested materials, the specimen surfaces before the wear test, the central surface of the wear scar, and the results of the elemental analysis. Red arrows indicate microcracks, green arrows show pull‐out filler particles, black arrows point to fractures of filler particles, and the yellow arrow shows a bulk fracture.

Figure 11 shows scanning electron micrographs of the composite samples, highlighting notable differences in filler shape and size distribution. Spherical nano‐sized fillers are predominant in the EA composite, while the NS composite contains spherical micro prepolymer fillers along with irregular fillers. The EBF and CM composites feature irregular fillers of various sizes and sharpness. Before testing, the surface appears smooth with minimal visibility of particulate fillers, which aligns with the specimen preparation method: the surfaces were covered and polymerized against a glass microscope slide. The wear scar area reveals particle loss paths across all tested composites. EBF, NS, and CM exhibit deeper microcracks at the micron‐sized filler‐resin interface, whereas EA shows minor microcracks. Partial damage to larger filler particles is noticeable in CM. Consistent with the SR results, larger exfoliated filler particles correspond to a wider SR range. EA demonstrates bulk loss rather than individual‐particle loss, which also aligns with the SR findings. The elemental composition results confirmed the data provided by the manufacturer for each material type. The filler size distributions, based on the SEM images, are presented in Table 2.

**Table 2:** The filler size distribution calculated from SEM micrographs according to the tested materials.

	Estelite asteria	Enamel biofunction		Neospectra		Clearfil majesty	
Size in nm	Nanoscale filler	Nanoscale filler	Submicron size filler	Nanoscale filler	Micron size filler	Nanoscale filler	Micron size filler
Mean	150	40	243	340	13268	30	1135
SD	38	12	165	187	7468	12	832

## 4. Discussion

We aimed to compare the two‐body wear resistance of universal nanocomposites proposed as enamel replacement materials for both indirect and direct applications. The four nanocomposites evaluated represent different filler technologies, each characterized by its filler type: round particles, prepolymerized fillers (PPFs), irregularly shaped fillers, and nanoparticle clusters within distinct resin matrices. Specifically, NS contains spherical PPF (generated via SphereTEC technology) and nonagglomerated barium glass and ytterbium fluoride fillers. EA is composed of spherical silica–zirconia fillers approximately 200 nm in size. CM features a complex ultrastructure, with larger fillers surrounded by nanosized pulverized filler particles. EBF, a BisGMA‐free composite, utilizes nanosized particles in an agglomerated form to enhance biocompatibility. Among the materials tested, CM composite demonstrated significantly higher two‐body wear resistance (as indicated by MVL and MWD data) compared to the others, which yielded very similar results (Figures 7 and 9). Thus, the first null hypothesis was partially accepted.

A significant difference was also observed in VH and SR data between CM and the tested RBC (Figures 5 and 10), thereby rejecting the second null hypothesis.

A dental restoration should have a wear rate similar to that of natural human enamel [28, 29]. Many direct and indirect additive materials have been thoroughly studied for posterior restorations [1, 5, 6, 9, 29]. Based on earlier research, only Type III gold alloy was considered suitable for occlusal rehabilitation because of its favorable wear properties and marginal adaptation. However, as esthetic demands increased, tooth‐colored materials became the preferred choice for restorations. In earlier in vitro studies HRi Function and EBF have shown wear resistance similar to Type III gold alloy, making them suitable for both direct and indirect clinical use [30, 31]. We chose EBF for this study due to its wear resistance.

In recent decades, the development of new RBCs has been driven by advances in filler technology. Fillers have been generated with smaller particles [32]. Additionally, new compositions such as prepolymer fillers [33], monomodal supranano fillers [34], and nanosized clusters [35] aim to enhance the overall characteristics of this material group. The filler morphology, which varies greatly, also significantly affects the filler load in RBCs and certain physical parameters [11, 14].

When measuring the DC, we assumed that if the polymerization rate does not vary across different matrix resin compositions, then the filler components will significantly influence the physical and mechanical behavior of the RBCs. The DC after thermocycling ranged from 66.72% ± 4.70% to 70.71% ± 3.26%, with no significant differences among the RBCs (Figure 4). Therefore, each tested composite exhibits a very similar trend in conversion rates.

The composite with the highest filler load by volume and weight (CM) showed a significantly higher VH [36] on the top and bottom surfaces compared with the other composites [37]. Consistent with earlier results [35, 36], thermocycling significantly reduced VH of all composites (Figure 5). The significantly higher VH of CM, both before and after thermocycling, can be attributed to its highest filler load and low water sorption [26]. The filler load has a substantial direct effect on VH [14], while water sorption is also associated with other factors such as the silanization of the fillers [15]. In addition, the filler composition and the filler technology [38] and filler morphology [14] can also influence VH. The similar VH values for EA and NS can be explained by their similar filler size range (0.1–0.3 μm) and filler weight percent (78%–82%); moreover, the NS composite also contains prepolymer fillers as well. Similarly, to CM, EBF contains irregular nano‐ and microsized particles. However, the aggregated nanosized fillers in EBF are associated with a lower filler volume percentage, which may explain the significant difference in VH between EBF and CM.

The VHR is calculated by dividing the VH of the bottom surface by the VH of the top surface. It correlates well with the bottom to the top DC of an RBC and is independent of the composite’s composition [39]. In our study, the VHR ranged from 94.4% (EBF) to 98.4% (NS), with no significant differences between the tested composites (Figure 6). If the VH of the bottom surface is at least 80% of the VH of the top surface, then, the applied layer is not thicker than the curing depth of the RBC [16, 40]. Based on our VHR results, we can conclude that both the applied layer thickness and the degree of cure of all tested composites were adequate for the evaluation.

To ensure that our results are comparable with previous studies, we applied a widely accepted two‐body wear test, namely, the Willytec simulator with the Ivoclair method settings [6, 18, 29]. The antagonist material and form may also affect the wear rate of the tested materials. Therefore, we used steatite as an antagonist with a diameter of 6 mm; this is acceptable to imitate the upper first molar palatal cusp during wear tests [41]. The 120,000 cycles we applied represent approximately 6 months to 1 year in the oral cavity [42]. Furthermore, before the wear test, we performed thermocycling to imitate the thermal changes and humid environment in vivo. The 10,000 cycles we applied represent approximately 1 year in the oral cavity [25].

Due to their reliability and repeatability, optical methods are suggested over contact profilometry methods to examine the wear surface in clinical studies [42, 43]. In vitro studies use computer‐aided design and computer‐aided manufacturing (CAD/CAM) 3D contact scanners [30], 3D laser devices, optical sensors, and/or a profilometry device [18]. At present, there is no consensus regarding whether mechanical or optical sensors are more suitable to measure wear surface parameters [44]. We used scanning white‐light interferometry [45], a high‐resolution approach, to determine the MWL and MWD. Our white‐light interferometer allows for the investigation of surface morphology, topology, and smoothness. It can display a 3D image of the sample’s surface as well as cross sections in planes perpendicular to the surface. The resulting profiles can be analyzed, and specific distances measured (Figure 8). Various built‐in surface texture parameters, such as RMS roughness and others, can be determined for the entire surface. CM with the highest filler load—resulting from the combination of pulverized nanofillers around larger microfillers—exhibited the lowest MVL and MWD, which is consistent with previous findings [7, 36]. The high filler fraction in homogeneously distributed structures may be associated with improved wear resistance [21, 46].

It was [47] reported that pretest SR does not influence the wear rate. However, posttest wear SR is associated with the surface characteristics of the tested material, which is consistent with our SR and SEM findings (Figures 10 and 11) [48]. Based on our profilometry results and consistent with the wear surface characteristics, CM exhibited the lowest Ra values, although the difference was significant only at the 10% level. A higher SR correlates with a higher wear rate. Our SEM analysis revealed scratches on the surface aligned with the applied load direction, matching the irregularity of the wear surface (Figure 11). The absence of filler dislodgement, protrusion, or other irregularities suggests a strong resin–filler bond. The composite surfaces also showed signs of plastic deformation, abrasive, and fatigue wear [9, 21]. The particular fillers may respond differently under load. They can deform, fail, or crack. Nanoclusters, in particular, behave quite differently from discrete filler particles. Under lower forces, nanoclusters might deform or become dislodged, and sometimes exhibit multiple fractures, a phenomenon not typical of particulate fillers. Depending on their composition, nanoclusters show significant variability in behavior, which can influence the physical parameters of an RBC [35]. Furthermore, the space occupied by the exfoliated particles may decrease due to the loss of the abraded resin matrix, as observed in our SR results. The SR has potential effects on esthetics, plaque retention, and wear behavior. A rougher surface leads to greater antagonist wear due to an increased coefficient of friction [49]. In an in vitro study, dislodged filler particles may remain on the surface and act as a third medium between the antagonist and the composite surface, or the water bath may wash them away in the testing chamber.

The unique characteristics of the resin matrix, the silanized interface, and the strong interactions among fillers influence the behavior of polymerized RBC. As previously reported, smaller filler sizes and higher volumetric filler loads have a beneficial effect on wear properties [50]. Earlier, Manhart et al. [51] highlighted that interparticle spacing in the resin matrix can be maintained by densely packed fillers [15, 52]. However, stable bonds between the fillers and the resin matrix also play an essential role in wear resistance [46]. Our SEM examination revealed distinct wear surface characteristics between nanofill and hybrid composites following the two‐body wear test (Figure 11). Nanosized particles, lacking the protective function of larger microsized fillers, tended to detach from the surface. Remnants of the separated filler and matrix mixture accumulated as a thin film on the wear surface. Additionally, the detachment of larger fillers may be linked to bulk surface loss [36]. Rigid, irregular fillers may concentrate stress at the silanized interface under applied force, ultimately leading to resin deformation and filler dislodgement [1, 21], while a harder matrix may retain the filler components more effectively (Figure 12) [53].

Figure 12The different surface wear mechanisms of the tested composites. (a) The spherical filler components and resin matrix are prone to abrasion caused by the antagonist. (b) The inorganic filler particles can protect the surrounding resin matrix from wear—similar to a nanohybrid composite with micron‐, submicron‐sized fillers, and nanofiller clusters. (c) After light curing, prepolymer fillers do not function as distinct filler components due to their mixed composition. (d) Tightly packed larger fillers combined with pulverized nanoparticles effectively protect the resin matrix, although wear may occur on the filler particle surfaces themselves. The loosening and detachment of these fillers lead to surface irregularities and increased roughness.

**Figure:**
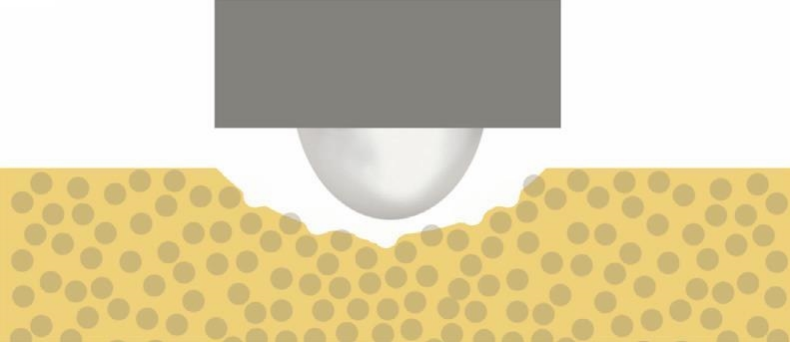
(a)

**Figure:**
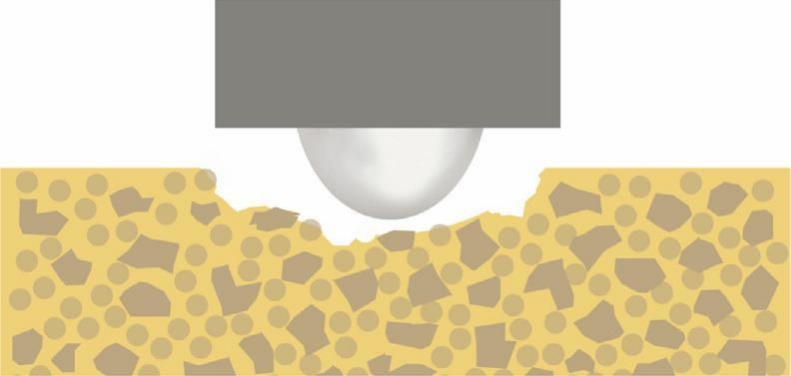
(b)

**Figure:**
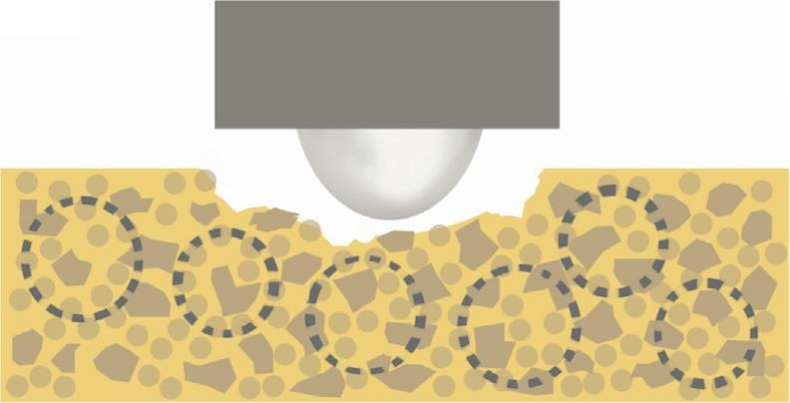
(c)

**Figure:**
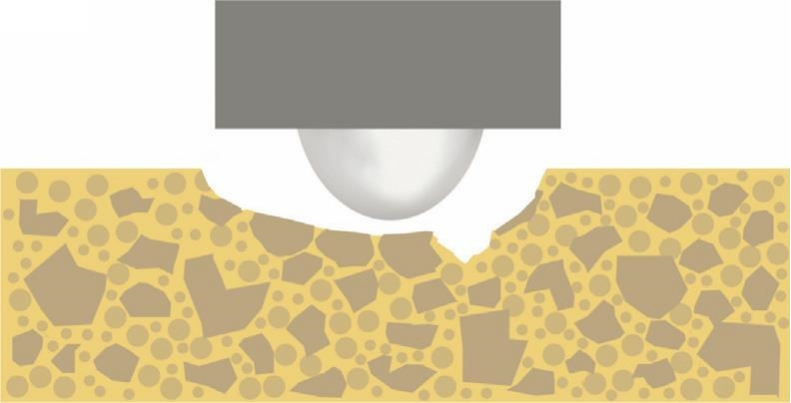
(d)

The composite with homogeneously distributed nanofillers exhibited similar wear resistance to composites containing prepolymers or nanofiller clusters. The spherical filler components and resin matrix are prone to abrasion caused by the antagonist (Figure 12a). After light curing, prepolymer fillers do not function as distinct filler components due to their mixed composition (Figure 12c). Therefore, only the inorganic filler particles can protect the surrounding resin matrix from wear—similar to a nanohybrid composite with micron‐, submicron‐sized fillers, and nanofiller clusters (Figure 12b). Tightly packed larger fillers combined with pulverized nanoparticles effectively protect the resin matrix, although wear may occur on the filler particle surfaces themselves. The loosening and detachment of these fillers lead to surface irregularities and increased roughness (Figure 12d).

Considering the composition of the fillers, the size range of the filler fraction, and the distribution of nano‐ and micro‐fillers in the resin matrix, and the volume percent, the variability of these parameters among the tested commercial products made it difficult to obtain accurate predictions based on composition [26, 54]. The wear behavior of the tested RBCs depended on the material, consistent with findings from a previous study [50, 55], and can be attributed to the different technologies used by the companies manufacturing the RBCs [15, 21] and the various arrangements of the filler particles. It was [56] reported that small particles provide a microprotective effect when they are well dispersed. In contrast, agglomerated forms are less effective against wear due to altered interparticle spacing. CM contains pulverized nanoparticles, while in EBF, the nanofillers are agglomerated to improve biocompatibility. This structural difference may explain the distinct wear behavior observed in EBF compared to CM (Figure 11).

Considering a previous classification for RBCs [26] and our results, the parameters of the compact CM composite differ from those of the low‐fill EA, NS, and EBF composites. CM, with the highest filler volume percentage, exhibited the highest VH and significantly lower MVL and MWD than EA, NS, and EBF. However, the interactions among the resin matrix, silane coupling agent, and filler particles are also significant and vary with each composite. EBF has a wear rate comparable to enamel [30], making EBF an appropriate reference material in the present study. EA and NS showed no significant differences in MVL or MWD. Therefore, EBF, EA, and NS exhibited similar wear behavior. In this study, we applied a 50 N force, which is consistent with the physiological bite force [57]. CM showed a lower wear rate compared with the other tested RBCs; therefore, it is worth considering its application in patients with a higher bite force.

The main limitation of our study was that we only used the two‐body wear test and did not assess antagonist wear. Additionally, the heights of the tested samples varied according to the manufacturers’ instructions.

## 5. Conclusions

Considering the limitations of our study, the following results were obtained:

1. A significant difference in two‐body wear resistance was observed among nanocomposites with varying microstructures. CM exhibited a notably lower MWD compared to the other tested composites.
2. VH showed a correlation with the wear behavior of the nanocomposite with different microstructures. CM had the highest VH and demonstrated better wear resistance compared with the other tested RBCs, EBF, EA, and NS exhibited similar wear behavior.
3. The combined effect of having the highest volumetric filler load (82 vol%), strong bonds between larger filler particles and the resin matrix, and the protective role of dispersed nanofillers may explain CM’s superior wear resistance.

Significance: VH and wear behavior are significantly influenced by nanocomposite microstructure, including filler composition, size, and distribution.

## Funding

This research work is a part of “Project Number TKP2021‐EGA‐20 has been implemented with the support provided by the Ministry of Culture and Innovation of Hungary from the National Research, Development and Innovation Fund, financed under the TKP2021‐EGA funding scheme.”

## Disclosure

All authors have read and approved the manuscript.

## Ethics Statement

The authors have nothing to report.

## Conflicts of Interest

The authors declare no conflicts of interest.

## Data Availability

The data that support the findings of this study are available upon request from the corresponding author. The data are not publicly available due to privacy or ethical restrictions.
